# Curating and comparing 114 strain-specific genome-scale metabolic models of *Staphylococcus aureus*

**DOI:** 10.1038/s41540-021-00188-4

**Published:** 2021-06-29

**Authors:** Alina Renz, Andreas Dräger

**Affiliations:** 1 Computational Systems Biology of Infections and Antimicrobial-Resistant Pathogens, Institute for Bioinformatics and Medical Informatics (IBMI), University of Tübingen, Tübingen, Germany; 2 Department of Computer Science, University of Tübingen, Tübingen, Germany; 3 Cluster of Excellence ‘Controlling Microbes to Fight Infections’, University of Tübingen, Tübingen, Germany; 4 German Center for Infection Research (DZIF), Partner Site Tübingen, Tübingen, Germany

**Keywords:** Computer modelling, Biochemical networks, Molecular medicine

## Abstract

*Staphylococcus aureus* is a high-priority pathogen causing severe infections with high morbidity and mortality worldwide. Many *S. aureus* strains are methicillin-resistant (MRSA) or even multi-drug resistant. It is one of the most successful and prominent modern pathogens. An effective fight against *S. aureus* infections requires novel targets for antimicrobial and antistaphylococcal therapies. Recent advances in whole-genome sequencing and high-throughput techniques facilitate the generation of genome-scale metabolic models (GEMs). Among the multiple applications of GEMs is drug-targeting in pathogens. Hence, comprehensive and predictive metabolic reconstructions of *S. aureus* could facilitate the identification of novel targets for antimicrobial therapies. This review aims at giving an overview of all available GEMs of multiple *S. aureus* strains. We downloaded all 114 available GEMs of *S. aureus* for further analysis. The scope of each model was evaluated, including the number of reactions, metabolites, and genes. Furthermore, all models were quality-controlled using MEMOTE, an open-source application with standardized metabolic tests. Growth capabilities and model similarities were examined. This review should lead as a guide for choosing the appropriate GEM for a given research question. With the information about the availability, the format, and the strengths and potentials of each model, one can either choose an existing model or combine several models to create models with even higher predictive values. This facilitates model-driven discoveries of novel antimicrobial targets to fight multi-drug resistant *S. aureus* strains.

## Introduction

*Staphylococcus aureus* is an opportunistic pathogen that asymptomatically and permanently colonizes the nose of up to one third of the human population^1^. It is a commensal of the mucosae and the human skin, but can also cause severe infections with high morbidity, mortality, and healthcare-associated costs^2^. Methicillin-resistant *S. aureus* (MRSA) is one of the most successful modern pathogens^3^. In 2017, the WHO published a priority pathogens list for the research and development of new antibiotics. Among the clarithromycin-resistant *Helicobater pylori* and the vancomycin-resistant *Enterococcus faecium*, *S. aureus*, especially the methicillin-resistant *S. aureus* (MRSA), vancomycin intermediate (VISA), and vancomycin resistant strains (VRSA), are high priority pathogens^4^.

*Staphylococcus aureus* bacteremia (SAB) is a common infection^5^. The incidence rate ranges from approximately 20 cases per 100,000 persons per year in Canada^6^ to approximately 50 cases per 100,000 persons, inferred from the United States surveillance data^7^. The higher incidence rate might be due to the greater burden of MRSA^5^. SAB can be classified into three categories: (1) Hospital onset of health-care associated infections, e.g., nosocomial; (2) Community onset of health-care associated infections, and (3) community acquired infections^8^. Besides SAB, *S. aureus*, and especially MRSA, is the leading cause of endocarditis, bone and joint infections, skin and soft tissue infections, and further hospital-acquired infections^3^. A study from 2013 revealed over 80,000 invasive infections and 11,000 deaths per year due to MRSA in the United States. Compared to the previous years, the number of invasive MRSA infections declined slightly^9^. Unfortunately, the rate decline of MRSA infections has recently slowed down according to the “Morbidity and Mortality Weekly Report” of the Centers for Disease Control and Prevention^10^, while the number of methicillin-susceptible *S. aureus* (MSSA) bloodstream infections even slightly increased. In 2017, nearly 120,000 *S. aureus* bloodstream infections and 20,000 associated deaths occurred in the United States^10^. Hence, strategies for preventing infections inside and outside acute care settings are required to further reduce the amount of invasive MRSA infections.

The transmission of *S. aureus* in general, and MRSA in particular, is facilitate by the long persistence time of *S. aureus* colonization. Nearly any item with skin contact can serve as fomes. In a hospital setting, this can include coats and clothes from doctors and nursing staff, pens, and mobile devices, such as cell phones^3^. Studies also suggest that infecting *S. aureus* isolates also persist in households three months after skin infections^11^. Even across and within athletic fitness facilities, *S. aureus* is found on different surfaces, including weight plates and treadmill handles^12^.

Besides the challenge of controlling *S. aureus* colonization in multiple environments, *S. aureus* strains evolve and adapt to different environments due to variability in diversity, mobile genetic elements (MGEs), and accumulation of mutations^13–15^. Mediators of virulence, immune evasion, and antibiotic resistance are commonly found within the accessory components of the *S. aureus* genomes, consisting of MGEs with pathogenicity islands, chromosomal cassettes, transposons, plasmids, and bacteriophages. Compared to the core genome, the accessory genome is more variable and also often more strain-specific^3^. MGEs in *S. aureus* can carry antibiotic resistance genes for resistances against penicillin, trimethoprim, erythromycin, clindamycin, and tetracyclines^15^. However, strains not only evolve and develop antibiotic resistances, they even replace each other within the same host^14^.

To fight *S. aureus* infections, several new antimicrobial and antistaphylococcal drugs have been developed recently^3,13^, including oritavancin and ceftaroline^16,17^. Despite the development of new antibiotics, *S. aureus* in general, and MRSA in particular, remains a prominent pathogen with persisting high mortality^3^. Since *S. aureus* will continue to evolve and develop new resistances^13^, the research on *S. aureus* and the development of new antimicrobials is of urgency to fight *S. aureus* infections.

One possibility for the identification of novel targets for antimicrobial therapies is the use of genome-scale metabolic models (GEMs). Advances in high-throughput techniques and whole-genome sequencing facilitate the construction of GEMs^18,19^. They are reconstructed based on information from genome sequences and experimentally obtained biochemistry^19,20^. With this information, stoichiometry-based and mass-balanced metabolic reactions can be formulated using gene-protein-reaction associations (GPRs). These stoichiometry-based GEMs can predict metabolic flux values within the constructed network^21^ and optimization techniques. Optimization techniques, such as flux balance analysis (FBA), use linear programming^20^. Recent advances in the reconstruction of GEMs and the fast analysis and integration of omics data enabled metabolic studies with model-driven hypotheses and context-specific simulations^22,23^. Among the multiple applications of GEMs is the drug targeting in pathogens and the modeling of interactions among multiple cells or organisms^20^. These approaches could be used to investigate and develop novel antimicrobials or antistaphylococcals. However, depending on the pathogen and strain, various models of *S. aureus* strains might be required to investigate the best antistaphylococcal target for a certain *S. aureus* strain.

In this review, we present all currently available GEMs of *S. aureus* from various databases. The available models were compared regarding their scope, their availability, their format, and their immediate usability. For various reasons, some of the models required revisions, such as converting spreadsheet file formats to SBML^24^ or ensuring the syntactic validity of SBML files. After having all models available as syntactically valid SBML files, their growth-capabilities, their predictive value, and the similarities between the various models were investigated. This review gives an overview of the available models and their properties to identify the appropriate model for a specific research question.

## Model overview

### Introduction of the models

Databases such as BiGG^25^ or BioModels^26^ comprise a variety of genome-scale metabolic models. Together with models from other databases and supplementary information from scientific publications, a large number of genome-scale metabolic models of *S. aureus* is available: The BioModels database contains two models of *S. aureus* by Becker et al.^27^ and Heinemann et al.^28^, both build in 2005. The BioModels database also harbors the models created within the Path2Models project^29^. In this project, 33 whole genome metabolism models of *S. aureus* were automatically created and curated between 2012 and 2013^29^. The BiGG Models Database contains two GEMs of *S. aureus*: the already mentioned model by Becker et al.^27^ and a recently published model by Seif et al.^30^ from 2019. Lee et al. published thirteen genome-scale metabolic reconstructions of multiple *Staphylococcus aureus* strains in 2009^31^. In 2016, Bosi et al.^32^ curated and published 64 genome-scale metabolic models of various *S. aureus* strains. Together with the *S. aureus* model published within the gut microbiota resource of the Virtual Metabolic Human (VMH) Database^33,34^, a total number of 114 genome-scale metabolic models of *Staphylococcus aureus* exists today.

**Fig. 1:**
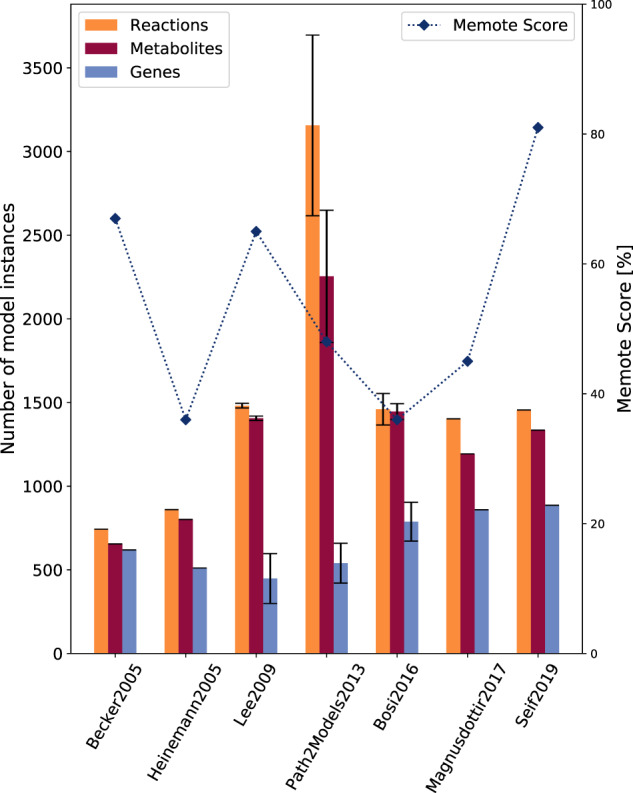
Properties of all available *S. aureus* models and their scopes. For all models, the number of reactions, metabolites, and genes in the model is illustrated. MEMOTE conducts standardized and community-maintained metabolic tests for quality control and quality assurance of genome-scale metabolic models (GEMs) and assigns the tested model a score ranging from 0 to 100%. Lee et al.^31^, Bosi et al.^32^, and the Path2Models Project^29^ published a collection of different *S. aureus* models. For the collections, the mean number of model instances is shown and the error bar indicates the standard deviation (s.d.).

All models were downloaded, tested, and evaluated using COBRApy^35^ and MEMOTE^36^. MEMOTE is an open-source software that contains a standardized and community-maintained set of metabolic model tests^36^. The overall MEMOTE score comprises information about annotations of metabolites, reactions, and genes, the inclusion of Systems Biology Ontology (SBO) terms, and the model’s consistency. Within the annotations sections, the presence and conformity of different database identifiers is evaluated. In the SBO term section, the annotation of model instances with appropriate SBO terms is assessed. The model consistency check comprises tests to evaluate the stoichiometric consistency, mass and charge balances, metabolite connectivity, and unbounded fluxes in default medium^36^. However, the MEMOTE score currently does not consider information about e.g., realistic growth rates, orphan or dead-end metabolites, stoichiometrically balanced cycles, or duplicated reactions. MEMOTE includes this information in its report but does not incorporate it into the calculated score. The number of model instances and their MEMOTE score are indicated in Fig. 1.

#### *i*SB619—GEM by Becker et al.

The first, initial draft of an *S. aureus* genome-scale reconstruction was curated by Becker and Palsson in 2005. They reconstructed the *S. aureus* strain N315 with 619 genes, 743 reactions, and 655 metabolites. The GEM was curated based on the key metabolic pathways in the Kyoto Encyclopedia of Genes and Genomes (KEGG) database^37^. Subsequently, The Institute for Genomic Research (TIGR) website^38^ was browsed for additional reactions. 91% of all reactions are linked with genes or open reading frames in so-called gene-protein-reaction associations (GPRs). This first-draft GEM is almost completely elementally and charge balanced. The biomass objective function was formulated based on the biomass data from *Bacillus subtilis*^39^ and substituted where necessary. It contains metabolites, such as amino acids, nucleotides, lipids, and cell wall constituents^27^. The first *S. aureus* GEM reached a MEMOTE score of 67% and is available as a file in SBML Level 3 Version 1^40^ format with flux balance constraints (fbc) extension^41^ and BiGG identifiers.

#### *i*MH551—GEM by Heinemann et al.

In the same year, the second genome-scale reconstruction of *S. aureus* was published by Heinemann et al. Both research groups curated the *S. aureus* strain N315 and used the KEGG^37^ and TIGR database^38^, together with literature for genome regions with limited sequence homology for gene function assignments. A new biomass objective function was specifically defined for *S. aureus* based on integration of literature data from a variety of different *S. aureus* strains. The biomass objective function was build upon the five polymer categories DNA, RNA, proteins, lipids, and cell wall components, and extended by pool solutes. The reconstruction includes 801 metabolites and 860 reactions that are based on 551 genes and simulates aerobic and anaerobic growth^28^. This *S. aureus* GEM reached a MEMOTE score of 35% and is also available as SBML Level 3 file with fbc extension. The genes are not included in the SBML file.

#### GEMs by Lee et al.

Lee et al. utilized the ERGO^TM^ bioinformatics suite^42^ and the KEGG ligand/reaction database^37^ to curate metabolic reconstructions of multiple *S. aureus* genomes. The DNA sequence and associated open reading frames (ORFs) or protein sequences were integrated into the ERGO genome database. ORFs were called via a combination of programs and annotated automatically or manually. BLAST was used to compute the protein similarities. Functional assignments, relationship computation, and pathway analyses based on existence of ortholog and protein family clusters led to automated metabolic reconstructions. Manual steps included the review of every gene in the genome, pathway curations, and the consideration and reconciliation of motif/domain database results for functional assignments. For identified missing steps within a certain pathway, Lee et al. searched for orthologs or published biochemical activities. For all complete, incomplete, or partial EC number annotations, associated reactions were identified via the ERGO pathway collections and KEGG database. Lee et al. used both biomass compositions from Becker et al.^27^ and Heinemann et al.^28^ for their analyses. On average, the thirteen *S. aureus* reconstructions included 1476 ± 14 reactions and 1406 ± 11 metabolites. All models are available as Excel spreadsheet files with KEGG identifiers.

#### GEMs from Path2Models

More than 140,000 freely available and automatically generated mathematical models from pathway representations are available through the Path2Models project. KEGG^37^, BioCharta^43^, MetaCyc^44^, and SABIO-RK^45^ served as databases to generate three types of models, including genome-scale metabolic reconstructions. The pipeline for generating GEMs starts with the extraction of pathway data from KEGG^37^ and MetaCyc^44^. To reconcile the different metabolite and reaction identifiers, MNXref was used^46^. MNXref was further used to define default metabolite formulas and charge states. It allowed the mapping to different databases for a semantical annotation in accordance with the Minimal Information Required In the Annotation of Models (MIRIAM) guidelines^47^. To all GEMs, a default biomass objective function containing all 20 amino acids, RNA and DNA nucleotide precursors, glycogen, and ATP was added. Between 2012 and 2013, 33 *S. aureus* GEMs were curated with the help of this pipeline, including one bovine strain. This strain had 6110 reactions, 4416 metabolites, and 1198 genes. The other *S. aureus* GEMs have on average 3064 ± 103 reactions, 2186 ± 75 metabolites, and 519 ± 12 genes. All models have a MEMOTE score of 48% and are available at the BioModels database as SBML Level 2 files^48^ with mixed nomenclature.

#### GEMs by Bosi et al.

In 2016, Bosi et al. constructed 64 GEMs of different *S. aureus* strains. They started by extending and adding content from KEGG^37^, Model SEED^49^, and MetaCyc^44^ to the *S. aureus* N315 model *i*SB619 by Becker et al. This manually curated model was used as reference for other *S. aureus* strains. Shared genes and reactions were identified and subsequently, strain-specific metabolic content available from KEGG^37^, Model SEED^49^, and BioCyc^50^ was manually added to the strain-specific GEMs. Since an *S. aureus* biomass composition was not available, the biomass objective functions from Becker et al.^27^ and Heinemann et al.^28^ were combined and *S. aureus*-specific data regarding the fatty acid composition in the biomass were used to adjust the biomass objective function. A gap-filling step further refined the models. On average, the models have 1460 ± 94 reactions, 1446 ± 47 metabolites, and 788 ± 116 genes with an average MEMOTE score of 36 ± 1%. All models are available as SBML Level 3 files^51^ with fbc extension and BiGG nomenclature.

#### GEM by Magnúsdóttir et al.

To elucidate the role of microbial communities in human metabolism and health, Magnúsdóttir et al. semi-automatically generated genome-scale metabolic reconstructions of 773 human gut bacteria, including *S. aureus* USA300-FPR3757^33^. By using a comparative metabolic reconstruction method that propagates refinements from one metabolic reconstruction to others, the model quality of all 773 models was improved. The basis for each reconstruction were draft GEMs from Model SEED^49^ and KBase^52^ including gap-filling, refinement via rBioNet^53^, and quality control and quality assurance testing. Further refinement steps included the verification of reaction directionalities as well as mass and charge imbalances. The reconstructions were extended by gut-microbiota specific subsystems and central metabolic subsystems, and anaerobic growth was enabled. Leak tests and the removal of infeasible flux loops further refined the model. The *S. aureus* model contains 1403 reactions, 1193 metabolites, and 859 genes, and reached a MEMOTE score of 45%. It is available as SBML Level 3 file with fbc extension and VMH nomenclature.

#### *i*YS854—GEM by Seif et al.

Seif et al. manually reconstructed a comprehensive genome-scale metabolic model of *S. aureus* USA300 str. JE2 containing 886 genes, 1455 reactions, 1335 metabolites, and 673 three-dimensional protein structures. The GEM was build upon one of the reconstructions of Bosi et al.^32^. Extensive and detailed manual curation was supported by literature reviews and network evaluations. The initial model was extended by an updated biomass objective function. Model instances, such as genes, reactions, and metabolites, were enriched with cross-references and metadata. More than 50 metabolic sub-modules were examined, curated, and added to the GEM, together with over 200 confidence scores and 300 references. By this manual curation, 569 new metabolic processes, 214 new ORF assignments and 207 new metabolites were added. Experimental validation of the model revealed an 85% agreement with gene essentiality data and 68% agreement with experimental physiological data^30^. A model evaluation with MEMOTE revealed with 81% the highest MEMOTE score of all tested models. The model is available as SBML Level 3 file with fbc extension and BiGG identifiers.

### Presence of strains

**Fig. 2:**
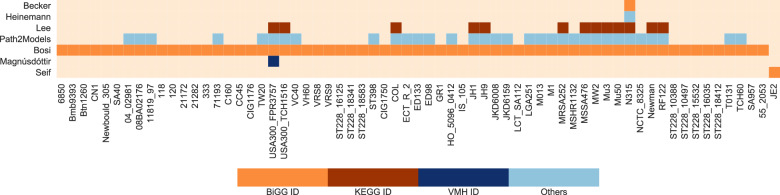
Occurrence of models for *S. aureus* strains. Sixty-five different strains of *S. aureus* are available over the seven publications. Some *S. aureus* strains, such as the *S. aureus* strain USA300-FPR3757, occur in several publications, for other strains, only one publication is available. The colors indicate the utilized metabolite and reaction identifiers in the respective models. Models with similar or same identifiers can be compared more easily.

The 114 currently available GEMs divide into 65 different *S. aureus* strains. In Fig. 2, the diverse *S. aureus* strains and their occurrence in the different publications is illustrated. Some strains, such as USA300-FPR3757 or N315 occur several times in different databases. Others, like the GEM for *S. aureus* strain JE2 occur only once in literature so far. The colors indicate the metabolite and reaction identifier in the respective model. Among the five models of the strain N315, two models exist that both carry BiGG identifiers. Models with same identifiers can be compared more easily than models with discriminating identifiers. Thirteen *S. aureus* strains occur at least in three different databases or publications with varying identifiers.

Due to the vast amount of different *S. aureus* strains, we elucidate only the strains that are shared over multiple databases. As already mentioned, the GEMs of the *S. aureus* strain N315 are the most prevalent. This strain was isolated from the pharyngeal smear of a Japanese patient in 1982^54^. It is a methicillin-resistant *S. aureus* (MRSA). The only effective antibiotic against it was vancomycin. However, in 1997, a vancomycin-resistant MRSA strain, Mu50, was discovered in a Japanese infant with a surgical wound infection^54^. The closely related strain Mu3 is a hetero vancomycin-intermediate MRSA strain. Strains with heterogeneous vancomycin resistance can spontaneously produce cells with increasing resistance against vancomycin^55,56^.

The isolates JH1 and JH9 stem from a series of MRSA isolates obtained from a patient receiving extensive therapy. These strains are also vancomycin-intermediate *S. aureus*. The first isolate, JH1, was taken before the chemotherapy and was fully susceptible to vancomycin. The last isolate, JH9, from the end of the therapy showed decreased susceptibility to vancomycin^57^.

The *S. aureus* strains of type USA300 are clones of the community-acquired MRSA^58,59^. It causes invasive infections in children and adults in the USA^58^, but also in Canada and Europe^59^. It is suggested that USA300 is more virulent than other community-acquired MRSA strains^58^. FPR3757 is a multidrug-resistant USA300 strain with acquired mobile genetic elements (MGEs) encoding resistance and virulence determinant that probably lead to enhanced pathogenicity^59^. The other USA300 isolate, TCH1516, also named USA300-HOU-MR, was isolated at the Texas Children’s Hospital in 2007. Significant differences to other MRSA strains lie within the plasmid content and the antibiotic susceptibility profiles^58^.

MW2 is another community-acquired MRSA isolate. It carries a wide range of virulence and resistance genes^60^. At the moment, more than fifteen different pathogenicity islands are identified in *S. aureus*. Interestingly, MW2 contains almost the same complement of pathogenicity islands as USA300-TCH1516^58^. In contrast, the *S. aureus* strain COL contains six pathogenicity islands, such as Mu50, but in different combinations^58^. COL is one of the first MRSA isolates from the early 1960s. It is a penicillinase-negative strain^61,62^. In contrast to the highly virulent MW2 strain, where virulence factors are found outside of prophages, fewer virulence factors are found outside of prophages in *S. aureus* strain Newman. This strain carries four integrated prophages and two large pathogenicity islands with important contributions for the pathogenesis. This *S. aureus* strain is susceptible to methicillin^63^.

As the Newman strain, the *S. aureus* isolate MSSA476 is a methicillin-susceptible clone. It is a community-acquired strain, such as MW2. It was isolated in 1998 and susceptible to most commonly used antibiotics, excluding penicillin and fusidic acid^64^. In contrast MRSA252 is a clinically important hospital-acquired MRSA lineage. It is genetically diverse to other *S. aureus* strains^64^.

*S. aureus* does not only infect humans, it is also the cause of a mastitis in cattle. Strain RF122 contains genomic features that distinguish the human and the bovine pathogens^65^.

Eight different *S. aureus* isolates belong to the South German clone lineage ST228. This clone spread over 10 years in a hospital in Switzerland. The isolates were collected between 2001 and 2008. The eight isolates represent the evolutionary history of the clone. As many others, it is an MRSA^66^.

## Model improvements

A variety of different *S. aureus* models from various strains is available. However, not all 114 downloaded *S. aureus* models were of the same quality: Some SBML files were syntactically invalid, others utilized an older SBML format, or were not available as SBML file at all. To provide a collection of usable and updated SBML models, we performed debugging and/or improvement steps on some of the models. Models with valid SBML files of the latest level were not improved. All debugging and improvement steps served the purpose of standardizing and annotating the models. No content changes were performed that affect model calculations.

### GEMs by Bosi et al.

The 64 *S. aureus* models by Bosi et al.^32^ were downloaded and evaluated using COBRApy^35^. The built-in validity check for SBML files returned a number of errors. In a first step, a pipeline for debugging the errors was created. All files lacked the XML declaration, which was added together with the XML version number and the encoding attribute. According to the SBML language specifications, metabolite, reaction, and model identifier need to fulfill certain properties^67^, e.g., model identifiers cannot start with a number. The identifiers were adapted according to the guidelines. The downloaded SBML file contained an empty compartment list, which was filled with the compartments during the debugging. As the compartment list comprises all cellular compartments in which metabolites and reactions occur, the different compartments were extracted from the metabolites’ information and subsequently incorporated into the compartment list. The charges, chemical formulas, and compartments of the models’ metabolites were adapted or added, where necessary. After these debugging steps, the models were exported as valid SBML files and evaluated with MEMOTE. The MEMOTE score of 36% in Table 1 is the score after these debugging steps, since MEMOTE requires a syntactically valid SBML file as input.

**Table 1:** Overview over the available *S. aureus* models.

Model	Year	Model count	Availability	Format	MEMOTE score	Initial growth	Curation
*i*SB619^27^	2005	1	BiGG & BioModels Database	SBML L3V1 with fbc	67%	✓	m
*i*MH551^28^	2005	1	BioModels Database	SBML L3V1 with fbc	36%	✓	m
Lee^31^	2009	13	Supplements	Excel spreadsheet file	65%		s
Path2Models^29^	2013	33	BioModels Database	SBML L2V4	48%		a
Bosi^32^	2016	64	Supplements	SBML L3V1 with fbc	36%	✓	s
Magnúsdóttir^33^	2017	1	Virtual Metabolic Human (VMH) Database	SBML L3V1 with fbc	45%	✓	s
*i*YS854^30^	2019	1	BiGG Models Database	SBML L3V1 with fbc	81%	✓	m

**Fig. 3:**
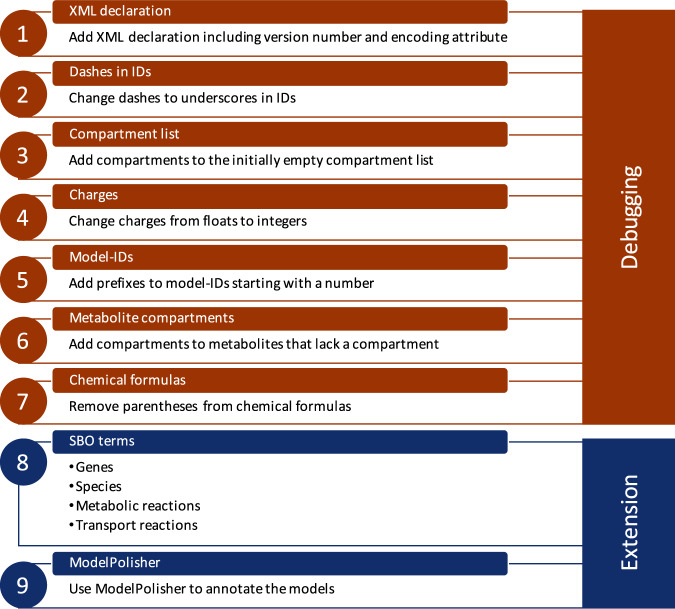
Debugging and extension steps in GEMs by Bosi et al. Not all the 64 SBML files downloaded from the supplement of Bosi et al. did directly pass the syntactic validation. In seven steps, the errors reported in the validity check were solved to receive valid SBML files. The valid files were then further extended with appropriate SBO terms for genes, species, and reactions. In a final step, annotations were added to the model using ModelPolisher^69^.

All GEMs were downloaded from the respective database or from the supplements of the publication. Their format and SBML version were determined. The initial growth was tested (indicated with the symbol ✓ in case of success) and the MEMOTE^36^ score was calculated for each model. The information for the models by Bosi et al. was determined after the debugging steps (see Fig. 3 steps 1–7). These debugging steps only served the purpose of generating valid SBML files. No additional improvements, which could increase the MEMOTE score, were performed at this point. The curation column indicates, whether the model was curated manually (m), automatically (a), or semi-automatically (s).

Since a pipeline for altering all 64 *S. aureus* GEMs already existed, we added further steps to the pipeline to extend the models with respect to their annotations. With the use of the Systems Biology Ontology (SBO), semantic information about model components can be provided. This information allows an explicit and unambiguous understanding of the components’ meaning^68^. For the model genes and metabolites, appropriate SBO terms were defined. Reactions were divided into metabolic and transport reactions, each receiving different SBO terms. Transport reactions were even further refined to active, passive, or co-transport with antiporters or symporters. After the assignment of appropriate SBO terms, further annotations were added using ModelPolisher^69^. ModelPolisher accesses the BiGG Models Database for the annotation and autocompletion of SBML models^69^. With the help of the ModelPolisher, additional metadata was incorporated for the different model instances. After those extensions, the MEMOTE score of the 64 GEMs increased on average to 83 ± 1%, which is an average improvement of 47%. The complete pipeline for debugging and extending all 64 models and saving them as valid SBML files is summarized in Fig. 3.

All debugging and extension steps served the purpose of making the models simulatable. Since reaction-sets, metabolite-sets, or gene-sets were not altered, the models’ simulation behavior is not affected. However, the models can now directly be used, as they are now all available as valid SBML files.

### GEMs by Lee et al.

The thirteen GEMs by Lee et al. were available as Excel spreadsheet. For all reactions and metabolites in the list, the respective information, such as reaction or metabolite name, or chemical formula was extracted from the KEGG database^37^, where available. Based on the information from the KEGG database and the Excel spreadsheet, a consensus model including all reactions was created. Both biomass objective functions from Becker et al.^27^ and Heinemann et al.^28^ were added to the consensus model, as well as exchange reactions for all extracellular metabolites. ModelPolisher^69^ was used for annotating the model. Based on this consensus model, the individual models of the thirteen *S. aureus* strains were curated: The strain-specific reactions listed in the Excel spreadsheet were added to the respective models, and the biomass objective function from Becker et al. was adapted strain-specifically. The KEGG database was browsed for the strain-specific gene identifiers. The models now include on average 491 ± 8 genes, except for *S. aureus* strain RF122, where no strain-specific KEGG gene identifier was available. Further annotations, such as KEGG annotations and EC-codes were added to the models. Despite manual effort, all thirteen models do not show growth for neither of of the biomass objective functions. The MEMOTE score for all models excluding the model for the *S. aureus* strain RF122 reached 66%. Since the GEM for the RF122 strain does not contain any genes, its MEMOTE score only adds up to 57%. Comparing the originally published models concerning model simulations and growth predictions is not possible, because only Excel spreadsheets with reactions and metabolites were available.

### GEMs from Path2Models

The 33 models from the Path2Models project are the only models of *S. aureus* that are still SBML Level 2 Version 4^70^. Since the fbc package is officially only available from Level 3, it is not yet integrated in the files. We updated all models to SBML Level 3 Version 1^40^ with the fbc package enabled using libSBML^71^. However, the original chemical formulas did not match the scheme that the official fbc package^72^ requires. In order to avoid creating syntactically invalid SBML files, all chemical formulas needed to be adapted according to the fbc specification^72^. The original chemical formulas can still be found in the notes field. This notes field further contained a variety of annotations from different databases, including BRENDA^73^, KEGG^37^, MetaCyc^44^, MetaNetX^46^, Rhea^74^, BiGG^25^, Reactome^75^, Model SEED^49^, Unipathway^76^, the Human Metabolome Database (HMDB)^77^, ChEBI^78^, and InChI^79^. All database annotations that can be found in the identifiers.org^47^ registry were transferred to the annotations, using identifiers.org uniform Resource Identifiers (URIs). The service identifiers.org provides directly resolvable identifiers from a multitude of different databases. The final and valid SBML files were evaluated using MEMOTE. The total score for the GEMs from the Path2Models project increased from 48 to 59% and all models are now available as SBML Level 3 files. Again, no changes on the reaction, metabolite, or gene content were performed, which would affect the model simulations.

## Model analysis

In the following section, we examined the available models for their predictive value and their similarity. As the growth behavior of *S. aureus* is reported in various defined media, the models’ capability of reflecting growth under these conditions indicates the predictive value of the model. Subsequently, the publications were checked for the inclusion of experimental data in the models or the verification of model-driven hypothesis. Additionally, the predictions of gene essentialities using different models are compared. In the last step, the models’ similarities were examined concerning their reaction and gene content.

### Growth capabilities

The growth of genome-scale metabolic models on different media is an important characteristic of a model’s capabilities and flexibility to reflect the organisms behavior in different environments. Since *S. aureus* is known to grow in a variety of different environments, its growth was simulated in chemically defined environments to investigate the model’s capabilities.

#### Chemically defined medium (CDM)

The CDM is a complete defined medium with 18 amino acids, two purines, and six vitamins and initially developed to study the slime production by coagulase-negative staphylococci^80^. It was used by Halsey et al. to study the amino acid catabolism in *S. aureus*^81^. Either no carbon source was added (CDM), or glucose (CDM_glc) or galactose (CDM_gal) was added to the medium. The growth of *S. aureus* strain JE2 is already computationally and experimentally validated and verified on CDM and its variants^30^.

#### Synthetic nasal medium (SNM)

The primary ecological niche of *S. aureus* is the human nose^82,83^. Krismer et al. developed a defined synthetic nasal medium (SNM) based on the composition of nasal fluid components determined by metabolomics^84,85^. This medium was initially developed to monitor the growth of *S. aureus* under similar physiological conditions as in the nose. Growth in this medium is experimentally verified for the *S. aureus* strains USA300 LAC and Newman. Since the medium is chemically defined, it can also be established in growth simulations in systems biology.

#### Gut medium

Already in the 1950s and 1960s, the intestinal colonization of *S. aureus* was reported^82^. Recent interest in the gut microbiome revealed and enlightened the relevant role and influence of *S. aureus* on the intestinal microbial ecology and diversity^83,86–89^. Intestinal colonization by *S. aureus* is, e.g., assumed to induce pseudo-membranous colitis and to change the gut microbial ecology^89^. Alterations in the composition of the gut microbiota can result in the development of chronic diseases, such as type 2 diabetes, colorectal cancer, and obesity^90^. Hence, studying the role of *S. aureus* in the context of the gut microbiome is of high relevance. Magnúsdóttir et al. generated 773 genome-scale metabolic reconstructions for 773 members of the human gut microbiome, including *S. aureus*. To simulate the growth in the gut, they chemically defined a medium according to experimental data. The medium definition was extracted from the *S. aureus* model created by Magnúsdóttir et al.^33^. Magnúsdóttir et al. validated two of the 773 genome-scale reconstructions experimentally, where *S. aureus* was not included. However, as their model grew in the defined medium, and *S. aureus* is reported to colonize the intestine, we inferred that growth should be possible.

#### SCFM

*S. aureus* does not only occur on the human skin, in the human nose^82,83^, or the nasopharyngeal tract^91,92^. It is furthermore observed in patients, especially in children, with cystic fibrosis (CF)^93^, an autosomal recessive disease. As one of the earliest and also most prevalent pathogens, *S. aureus* causes chronic airway infections in patients with CF^94^. To investigate the role of *S. aureus* and other associated pathogens, such as *Pseudomonas aeruginosa*^93^, Palmer et al. developed a synthetic cystic fibrosis medium (SCFM), mimicking the nutritional composition of the sputum of patients with CF by chromatographic and enzymatic analyses of the CF sputum. This medium was initially created to analyze the nutritional behavior of *Pseudomonas aeruginosa* in CF sputum^95^. Clinical isolates of *S. aureus* are reported to grow in SCFM^96^.

Since the thirteen models by lee et al. and the 33 models from the path2models project did not exhibit any growth in full medium, these models were not included in the analysis of growth capabilities. During the analysis, three of the models by Bosi et al. reported a low growth rate of 0.00186 mmol/(gDW × h) without any active exchange reactions (models sa_118, sa_gr1, and sa_lct). A positive growth rate without active exchange reactions can be an indicator for futile cycles and a necessity for manual verification and refinements.

**Fig. 4:**
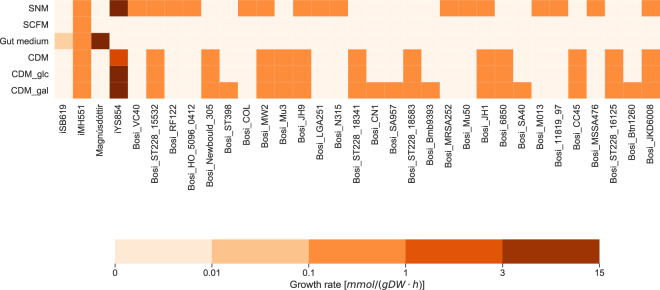
Growth rate of *S. aureus* GEMs in different media. All models with initial growth (see Table 1) were tested on the different media. For the GEMs by Bosi et al.^32^, the prefix “Bosi” was added to the model strain name. The other models are named according to their published model ID or, in case of the model from the VMH database, by the author. Media types are the synthetic nasal medium (SNM), synthetic cystic fibrosis medium (SCFM), gut medium, chemically defined medium (CDM), CDM with glucose (CDM_glc), and CDM with galactose (CDM_gal). Models that did not show growth in any of the tested media were excluded. The color-bar indicates the growth rate: the darker the color, the higher the growth rate of the model organism on the given medium.

Not all models by Bosi et al. were capable of growing on any of the tested media. In total, 33 out of the 61 remaining models were not able to grow on any of the tested media. This might be explained by the auxotrophies for amino acids and vitamins in several *S. aureus* strains observed by Bosi et al.^32^. None of the models by Bosi et al. grew on the SCFM or the gut medium. For SNM and the CDM compositions, different patterns emerged: ten strains, including N315, only grew on SNM, while six strains only grew on the CDM with galactose. Seven strains grew on all three variants of the CDM and the remaining five strain models grew on both the SNM and all CDM. The model *i*SB619 by Becker et al. only grew on the gut medium, while the model *i*MH551 by Heinemann et al. returned a positive growth rate for all tested media types. The model *i*YS854 exhibits growth on almost all tested media, except for the SCFM and the gut medium. It is comparable to the models by Bosi et al., with the difference of a higher growth rate. The model by Magnúsdóttir only grew on its own gut medium. In Fig. 4, the growth capabilities of the various *S. aureus* GEMs under different environmental conditions is illustrated.

### Presence of experimental data

Besides the correct prediction of growth in a defined environment, a model’s predictive value also increases when laboratory data is included or in silico observations are verified in laboratory experiments.

#### Automatically curated GEMs

The models from the Path2Models project were automatically constructed. Within automated reconstruction processes, the inclusion of experimental data for individual models is complicated. For this reason, the GEMs from the Path2Models project do not contain experimental data^29^. Moreover, the models are not simulatable and, thus, can also not predict any growth. Verification of model predictions is hence not possible.

#### Semi-automatically curated GEMs

Curating a collection of multiple GEMs is time and labor intense. Manual reconstruction would take a significant amount of time. Thus, the models from Lee et al., Bosi et al., and Magnúsdóttir et al. were constructed semi-automatically.

Lee et al. verified their models using gene essentially analysis and growth experiments of two models. They found literature evidence and experimental verification for six of the 44 identified genes that were essential in all strains in silico. The growth experiments supported their minimal-medium predictions^31^.

The models from Bosi et al. were examined for the correct simulation of already known auxotrophies. Furthermore, the predictions of the growth capability in the presence of spermidine, and the growth on chemically defined media were verified in laboratory experiments for several strains^32^.

The model from Magnúsdóttir et al. was curated based on literature-derived experimental data. However, it is not specified which experimental data is used exactly. Metabolic predictions of two of the 773 reconstructions were validated against experimental data^33^.

#### Manually curated GEMs

Becker et al., Heinemann et al., and Seif et al. manually curated their strain-specific GEMs. The in silico growth predictions of the model *i*SB619 in a minimal medium were compared to laboratory experiments. Becker et al. additionally predicted essential genes. As this was the first available GEM of *S. aureus*, no experimental data was available to compare the predicted essential genes with^27^. The model *i*MH551 was compared to available knowledge about auxotrophies in *S. aureus*. The model’s growth predictions under aerobic and anaerobic conditions were validated against available experimental evidence^28^.

The model *i*YS854 underwent the most experimental verifications compared to all other models. Its predictions are in 85% agreement with gene essentiality experiments. The in silico predictions of the catabolism of carbon sources are in 68% agreement with experimental physiological data. They compared the models’ growth predictions on various media with laboratory experiments, and performed extensive condition-specific GEM validation and evaluation in the presence and absence of glucose.

### Prediction of gene essentialities

Another indicator for the predictive value of a model is the correctness of predicted gene essentialities. The essentiality of a gene depends on the environment and the availability of nutrients. To identify essential genes in silico, each gene is individually knocked out in a so-called single gene deletion analysis and its effect on the growth rate is evaluated. This analysis, however, requires a model’s capacity to simulate growth in the investigated environment. As the models from the Path2Models project and Lee et al. did not show any initial growth (see Table 1), these models were excluded from the single gene deletion analysis. Additionally, this review aims to compare models from different sources. Since the models from the Path2Models project and Lee et al. were already excluded from this analysis, only two strains remain with more than one model: *S. aureus* USA300-FPR3757 and *S. aureus* N315. Two models from Bosi et al. and Magnúsdóttir et al. are available for the strain USA300-FPR3757, which can simulate growth. The model from Magnúsdóttir et al. contains gene identifiers that cannot be resolved within the PATRIC database^97^, leading to its exclusion from this analysis. With only one remaining model from Bosi et al., a comparison of predicted gene essentialities for the strain USA300-FPR3757 is not possible anymore.

Becker et al., Heinemann et al., and Bosi et al. curated models for the strain N315 simulating growth. The model from Heinemann et al., however, had to be excluded from the single-gene-deletion analysis as the model did not contain any GPRs and, thus, no genes. We downloaded the list of 302 essential genes for N315 from the Database of Essential Genes (DEG)^98^ and mapped all genes to the respective KEGG gene identifier. The medium is indicated as a rich medium in the DEG, but no further description of the chemical definition is given. Therefore, all exchange reactions were opened for the single gene deletion analysis.

The model from Bosi et al. predicted 117 essential genes, while the model from Becker et al. predicted 80. Of the 302 essential genes from the DEG, only 176 and 107 genes were present in the models from Bosi et al. and Becker et al., respectively. From the 117 predicted essential genes by Bosi et al., 27 (23.1%) were predicted correctly, while 90 (76.9%) of the predicted essential genes are not in accordance with the experimentally derived essential genes. Similarly, from the 80 predicted essential genes by Becker et al., 18 (22.5%) were predicted correctly, while 62 (77.5%) of the predicted essential genes are not listed in the DEG. One possible explanation for the similar predictions of essential genes is that the models from Bosi et al. are based on the model from Becker et al. The low number of true positive predicted essential genes could indicate further refinement potential of the two models.

### Similarities between models

The analysis of the growth capabilities implied a clustering of models with similar growth behavior, especially for the models by Bosi et al. To identify further similarities between the models, the reaction sets were compared. Mapping identifiers between different databases induces a bias, since a complete mapping is currently not feasible. Tools, such as ModelPolisher^69^, can be helpful for annotating and comparing models. However, these tools rely on cross-references in various databases, which holds some challenges: The tools can only search with the correct identifier; if a model, however, has identifiers not included in the database, the tools will not find any annotations for that model instance. One other challenge lies within the administration and topicality of the databases. Changes in one database might not be reported or updated in the cross-references of other databases, leading to erroneous allocations that would bias the result of the comparison.

#### Heat maps of reaction similarity

Since the models have diverging identifiers, we divided them into three different groups. The first group comprises the 33 models from the Path2Models project with consistently mixed identifiers The second group includes all thirteen models by Lee et al. with KEGG IDs. The third group includes all models with BiGG identifiers, namely all models by Bosi et al., as well as the models *i*SB619 and *i*YS854. Furthermore, this third group contains the model created by Magnúsdóttir et al. This model possesses VMH identifiers, however, those identifiers can easily be converted to BiGG identifiers since they bear a resemblance to the BiGG IDs. Within these groups, all reactions were listed and checked for their occurrence in the models. With this table of reaction occurrences, the Jaccard distance was calculated between all pairwise combinations of the models.

**Fig. 5:**
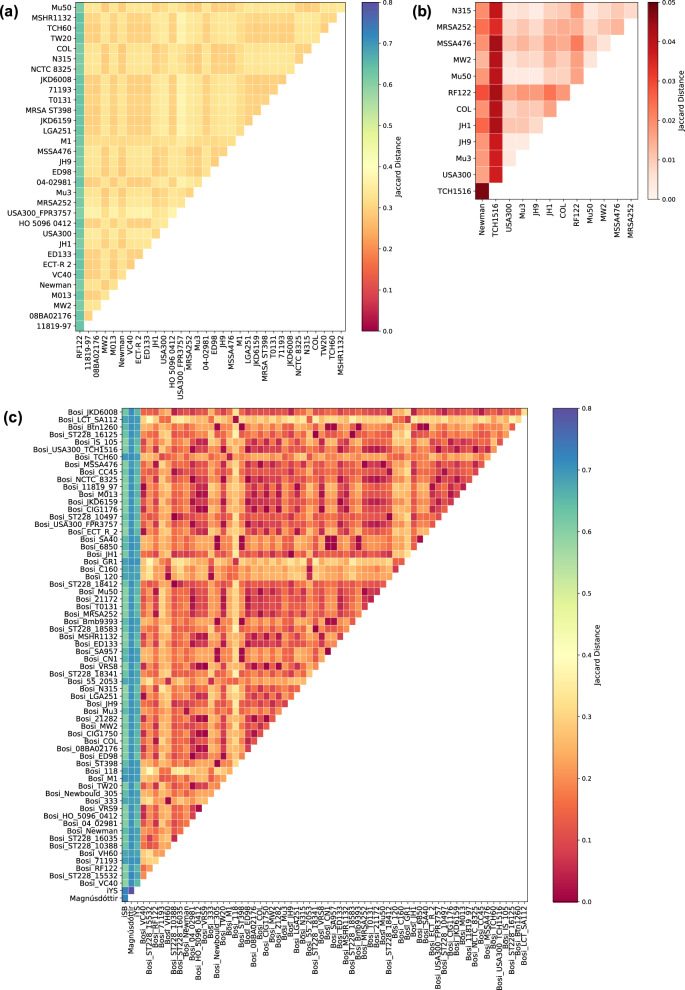
Model comparison based on Jaccard distance between reaction sets. The models were divided into three groups based on their metabolite and reaction identifiers: (**a**) has all models of the Path2Models project with consistently mixed identifiers, (**b**) has all models with KEGG identifiers (hence, all GEMs by Lee et al.), and (**c**) contains all models with BiGG identifiers. Within the three groups, all pairwise Jaccard distances were calculated based on the models’ reaction sets. The distances are displayed in the heat map. The color bar range is equal for (**a**) and (**c**) for better comparison. As the distances in (**b**) are much smaller, the color bar’s range was adapted.

With this distance matrix, the heat-map in Fig. 5 was created. The models *i*SB619, Magnúsdóttir, and *i*YS854 vary widely between each other and the models by Bosi et al. Within the Bosi models, clusters of more and less similar models can be identified (Fig. 5c). Such clusters are expected, as we assumed that genetically similar strains also lead to more similar GEMs, due to the gene–protein-reaction associations (GPRs). For example, the two closely related USA300 strains TCH1516 and FPR3757 have a distance value of 0.015, while the distance to one of the isolates of the ST228 lineage (ST228-16035) is 0.160. Strain MRSA252 is reported to be genetically diverse compared to other *S. aureus* strains. Its distance, however, to the USA300-TCH1516 strain is smaller (0.06) than the distance to the isolates of the ST228 lineage. Hence, the genetic differences between the different strains are not necessarily reflected in their respective GEMs so far.

The distances between the models with BiGG IDs (group three) ranged from 0 to 0.8, with the maximal distances between the models *i*SB619, Magnúsdóttir, and *i*YS854. The models by Lee et al., however, are more similar, indicated by the scaling of the color-bar that ranges from 0 to 0.05. The model of the *S. aureus* strain TCH1516 differs the most from all other models (Fig. 5b). Unlike the models from Bosi et al., the two USA300 strains (TCH1516 and USA300) do not cluster. They have a distance of 0.037. In contrast to the models of Lee et al., the strain TCH1516 does not stand out in the groups with BiGG IDs and the Path2Models models.

Most distances between the models from the Path2Models project (group one), ranged from 0.25 to 0.35. However, the model of strain RF122 protrudes with a mean distance of 0.62. This trend can also be observed in the heat-map of the models by Lee et al., but not as prominent as in Fig. 5a. One possible explanation is given in the taxonomy for the *S. aureus* strain RF122, which is an bovine mastitis-associated isolate with notable differences to human clones of *S. aureus*^99^. This difference is, however, not as obvious in the Models of Bosi et al. compared to the models of Lee et al. and the Path2Models project.

#### Venn diagrams of gene similarity

Despite significant effort to standardize and consistently annotate all models using different annotating tools, such as the ModelPolisher, or database requests for aliases from databases like BiGG or ModelSEED, a satisfying comparison of the reaction sets between different identifiers is still not possible. For example, for the models with KEGG identifiers from Lee et al., we could not use the ModelPolisher, as this annotation tool currently requires BiGG identifiers. For that reason, we browsed the BiGG Models Database locally for cross-references to KEGG identifiers. Unfortunately, 842 out of 1486 KEGG reaction identifier were not referenced at all in BiGG, 359 KEGG identifiers were not uniquely mapped to a BiGG identifier, and only 285 identifiers were uniquely mapped. We checked some of the non-referenced KEGG identifiers in the ModelSEED database for aliases but could not determine the respective identifiers.

For that reason, we looked at the gene content of the models. Most models used KEGG gene identifiers, regardless of the identifier database of the reactions and metabolites. As the different strains have strain-specific gene identifiers, the following analysis was conducted strain-wise. Strains with at least three models from various resources were taken into account (see also Fig. 2): For eleven strains, three models are available, for the strain USA300-FPR3757, four models are present in this collection, and for the strain N315, five models are available. However, the SBML file of the N315 model by Heinemann et al. does not include any genes. Thus, the model was excluded from the comparison. Same accounts for the RF122 strain-specific model by Lee et al., which also does not contain any genes. For this reason, the model was also excluded from the analysis. By that, the strain RF122 did no longer fulfill the criterion of at least three available models.

The gene sets from the remaining models were compared. As indicated, most models used KEGG gene identifiers, but not all. The model by Magnúsdóttir et al. included strain-specific and unspecific PATRIC identifiers^97^. With the help of the PATRIC ID mapping service, the respective KEGG gene identifiers were extracted. However, this was only feasible for the strain-specific identifiers. Despite significant effort, the unspecific identifiers could not be resolved, as no mapping scheme could be identified. Thus, from the 859 genes included in the Magnúsdóttir model, only 192 could be resolved to KEGG identifiers.

Model *i*SB619 contained new locus tags, whereas the KEGG identifiers correspond to the old locus tags. With the GenBank flat file (gbff)^100^ of *S. aureus* strain N315, the locus tags were mapped. For the 619 new locus tags 611 respective old locus tags, and thus KEGG identifiers, were extracted.

The models by Bosi et al. included mostly KEGG gene identifiers. Within the strains JH1 and JH9, the gene identifiers were truncated by the included word “DRAFT” to make them consistent with the actual KEGG identifiers. For example, the initial identifier SaurJH1DRAFT_0595 was truncated to the correct KEGG identifier SaurJH1_0595.

**Fig. 6:**
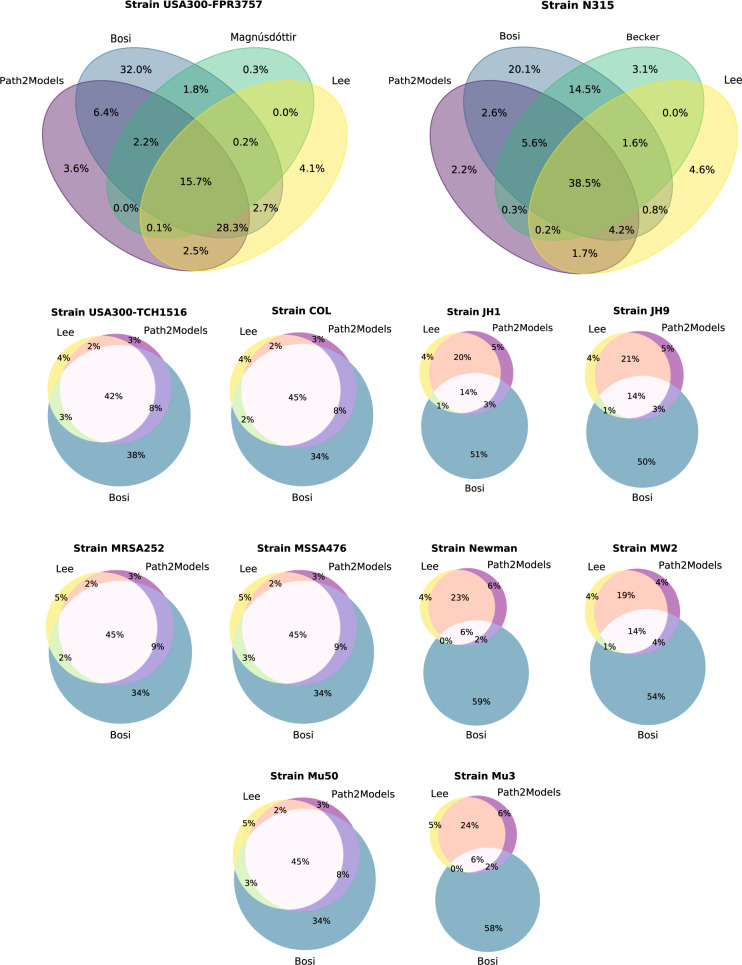
Strain-specific model comparison based on gene sets. For all models occurring in at least three different resources, the gene content was compared strain-specifically. After unifying the gene identifiers to KEGG IDs, Venn diagrams were created comparing the gene content. The models from Bosi et al. have, on average, the highest gene content, explaining the large fraction of genes occurring only in these models. The models by Lee et al. and the Path2Models project seem more similar, which could be explained by the fact that both are curated based on the KEGG database. Although all models in one Venn diagram (and thus, one comparison) represent the same strain, the models have differences, indicating the influence of the reconstruction method on the final model content.

After these mapping and adapting steps, the gene sets within the different strains from the different resources were compared, and Venn diagrams were created as shown in Fig. 6. Across all twelve comparisons, the models by Bosi et al. have the largest portion of genes that are solely reflected in these models. This number varies between 20.1% in the N315 strain and 59% in the Newman strain. As these models have the highest gene content on average with approximately 788 ± 116 genes per model, this seems apparent. The models from the Path2Models project have an average gene content of 519 ± 12 genes per model, and the models by Lee et al. contain 488 ± 149 genes on average. It was already mentioned that the gene identifiers from the JH1 and JH9 models by Bosi needed to be adapted. Despite this adaption, only half of the gene content is present in the other models as well. For the Newman, MW2, and Mu3 strains, we further analyzed the gene identifiers after these observed discrepancies between the gene contents with the models from the other two databases. These three strain-specific models from Bosi include non-strain-specific gene identifiers, which could not be mapped to the corresponding strain-specific gene identifier.

The models from Lee et al. and the Path2Models project are relatively similar concerning their gene content. Since both models are curated based on the KEGG database, this similarity is evident. The four models of the *S. aureus* USA300-FPR3757 strain have a gene content overlap of 15.7%. The model by Magnúsdóttir et al. has only 0.3% gene content that is not reflected in the other three models. However, one needs to keep in mind that many genes in the model are not strain-specific and could not be mapped and compared.

With these twelve gene content comparisons, we again calculated the Jaccard distance between the models from Bosi et al., Lee et al., and the Path2Models project. As already visible from the Venn diagrams, the models from Lee and the Path2Models project are most similar with respect to their gene content. They have a mean Jaccard distance of 0.288 ± 0.004. However, one might have speculated that the models are more similar based on the Venn diagrams. It needs to be highlighted that the Venn diagrams are calculated based on the gene content of all compared models. In contrast, the Jaccard distance calculates pairwise distances and, thus, only considers two models at once. For that reason, the models from Lee et al. and the Path2Models project are still the most similar ones, but their identity might not be as large as first expected when looking at the Venn diagrams. The Bosi models have a mean distance to the Lee models of 0.666 ± 0.179 and to the Path2Models project models a mean distance of 0.616 ± 0.203.

Although the different models from the various databases reflect the same strain, the models have distinct diversities. This can be explained by the differences in the reconstruction process. How the model is curated seems to play a pivotal role for the final model and its model instances. Thus, the reconstruction method needs to be chosen carefully, and manual or semi-automated additions might be required.

### Decision guidance

With the vast amount of different strain-specific *S. aureus* models, the identification of the suitable GEM for a specific research question or purpose might become difficult. Table 2 gives an overview about the main features of the *S. aureus* GEMs. The features were assigned based on the strengths of the different models or model collections after the model improvement steps. If one is interested in simulatable models, the table guides the reader to the corresponding models. By combining different required features, the selection can be tailored. If one needs, e.g., a model with BiGG IDs that grows on different media, the models by Bosi et al. or the model *i*YS854 are suggested, depending on the desired strain. High MEMOTE scores indicate a high degree of annotations, which facilitates the re-usability and comparability of a model.

**Table 2:** Feature-based decision guidance.

Feature	*i*SB619	*i*MH551	Lee	Path2Models	Bosi	Magnúsdóttir	*i*YS854
Database				✓			
Simulatable models	✓	✓			✓	✓	✓
BiGG IDs	✓				✓		✓
Kyoto Encyclopedia of Genes and Genomes (KEGG) IDs			✓				
Growth on different media		✓			✓		✓
High MEMOTE score	✓		✓		✓		✓
Predictive value score	3	5	0	0	3–5	2	7

The main features of the *S. aureus* GEMs are listed and indicated with the symbol ✓ when present. The models are assigned to the features based on their strengths after the model improvement steps. A predictive value score was calculated as described in the section “Decision guidance”. With the help of the features and the predictive value score, one can identify the best suited model for the research question of interest.

A predictive value score was calculated based on the model analysis regarding their growth capabilities and the presence of experimental data. If a model was not simulatable, it received a predictive value score of 0. Otherwise, a score of 1 was added. For growth capabilities in one environment, a score of 1 was added; for growth in multiple environments, 2 was added. For every experimental verification procedure, such as growth verifications, auxotrophies, compliance with physiological data, or other experiments, a score of 1 was added. The prediction of essential genes was not included in this score, as this analysis was only conducted for two models. By this scheme, the model *i*YS854 had the highest predictive value score of 7, followed by *i*MH551 and some models by Bosi et al. The models by Bosi et al. received a score between 3 and 5, as some models do not predict growth in any tested environment, while others do. As the models from Lee et al. and the Path2Models project are not simulatable, they received a predictive value score of 0. Models with high predictive value score and high MEMOTE score are recommended for further use, while models with low predictive value score might need further refinement and experimental verification before usage.

This table does not contain strain-specific information. Including the information from Figs. 2 and 4 will further guide the decision for a suitable model.

## Discussion

The analyses show that despite genomic and genetic similarities, GEMs of related strains are not necessarily similar to each other. This accounts for both models of the same strain curated by different research groups and to related strains curated by the same group. One example is the model from Magnusdóttir et al. with the *S. aureus* strain USA300-FPR3757 and the corresponding model from Bosi et al. Despite it is the same strain, the GEMs are quite different in their reaction content. In contrast, the two strain-specific models of the strains MRSA252 and USA300-TCH1516 by Bosi et al. are quite similar despite the genetic diversity of the strain MRSA252. This observation might have several reasons. The first, and probably most striking, reason is the incompleteness of the models. As high-quality genome-scale metabolic reconstructions require manual curation and evaluation^101^, and many models introduced in this review were created automatically or semi-automatically, some models might lack general or strain-specific reactions. This lack of required reactions is also visible when optimizing the flux distributions of the models. For multiple models, no growth could be simulated in FBA, not even in full medium. This was especially the case for the automatically curated models from the Path2Models project and the semi-automatically curated models from Lee et al. But also some of the semi-automatically curated models from Bosi et al. did not show any growth. Thus, a connection between automated or semi-automated curation and the functionality of the models seems to exist. However, automated or semi-automated curation does not necessarily result in poor growth prediction, especially when the basis for the (semi-) automated processes underwent significant manual curation. The other models from Bosi et al. showed growth on up to four different media. The semi-automatically constructed model by Magnusdóttir et al. could be simulated on one medium, which is also the case for the manually curated model *i*SB619. Furthermore, some of the *S. aureus* strains have plasmids carrying additional genes. For a strain-specific model, these additional genes need to be incorporated into the GEM as well. Especially the metabolic and transporter genes are relevant for the strain-specific model. The plasmid of the *S. aureus* strain N315, e.g., carries a gene for the cadmium resistance transporter CadD, which facilitates the export of cadmium ions and other cationic compounds^102^. Besides further proteinogenic genes, the plasmid of strain N315 also carries a gene for the penicillin-hydrolyzing class A *β*-lactamase enzyme. These two genes are, e.g., also present on the plasmid of the *S. aureus* strain USA300-TCH1516.

As explained previously, the challenge lies within the different reaction and metabolite identifiers. In this review, we additionally tried to annotate the GEMs further to simplify the comparison of models with differing identifiers. However, only approximately one third of all reactions and metabolites are annotated with identifiers of external databases. It is still challenging to find all cross-references for a particular metabolite or reaction in a specific database. For that reason, we additionally evaluated the gene content of the strain-specific models, as most models contained identifiers from the KEGG database. The gene identifiers from other databases were mapped to the KEGG identifiers. Again, a bias is introduced when identifiers are mapped between databases: On the one hand, not all identifiers can be resolved in the other database. On the other hand, some identifiers do not comply with the databases’ identifiers scheme and do not have annotations. This makes an automated mapping of several hundred identifiers infeasible. Extensive manual labor would be necessary to map these identifiers. The usage of consistent identifiers that comply with the database scheme and additional annotations is highly recommended and would simplify the re-usability, translatability, and comparability of models^103^. The comparison of the strain-specific models’ gene content confirmed that GEMs from different resources could vary, despite their genetic equality, highlighting the relevance of the curation process on the resulting GEM. This observation is even more explicit when comparing the models by Lee et al. and from the Path2Models project: both rely on the KEGG database. However, the models are not equal, as the two groups used different approaches for the curation of the models.

Missing reactions and strain-specific genes might also affect the growth behavior of a strain-specific model on a given medium. Only the model *i*MH551 showed growth on all tested media. Additional growth experiments for specific *S. aureus* strains can help to identify the missing growth capabilities of the model. The model’s ability to adapt to different environmental conditions is crucial to simulate an organism in silico. This is also reflected in the predictive value score, which was assigned to the models. Especially for models with a low predictive value score, additional experiments would help determine and also increase the predictive value of the model.

The models from Lee et al., the Path2Models project, Bosi et al., and Magnúsdóttir et al. are curated automatically or semi-automatically. Except for the models from Bosi et al., all models have a comparatively low predictive value score than the manually curated models. The models from the Path2Models project and Lee et al. have a score of 0. The low score from the Path2Models projects’ models might go back to the lack of experimental data in both the curation and verification process, thus highlighting its importance for predictive genome-scale metabolic reconstructions. The low score for the models from Lee et al. accentuates the importance of standardized GEMs, which allow re-usability. Although the models from Bosi et al. are curated semi-automatically, their predictive value scores are comparable high. They based their pipeline on a manually refined model and verified their predictions with experimental data. More experimental data accompany more knowledge. The latest model, *i*YS854 has the highest predictive value score, was manually curated, and extensively experimentally validated. The result of such a time- and labor-intensive work is a GEM with a high predictive value and a strong recommendation for future usage.

## Conclusion and outlook

In this review, all 114 currently available genome-scale metabolic models (GEMs) of *Staphylococcus aureus* were presented and evaluated. It serves as guide for the different available reconstructions in various databases, using differing metabolite and reaction identifiers. Some models originally comprise a large number of reactions, metabolites, and genes, after undergoing several manual curation steps and extensive annotating. Such models have a high MEMOTE score. The model with the highest MEMOTE score is the *i*YS854 model by Seif et al. Other models have a vast amount of reactions and metabolites, such as the reconstructions of the Path2Models project. Such models could, e.g., serve as information sources for the reconstruction or refinement of already existing strain-specific models. Based on the information regarding availability, model format, MEMOTE score, growth behavior, used database identifiers, predictive value, and similarities between models, together with a previously defined research question, the appropriate genome-scale reconstruction can be chosen from the vast amount of available GEMs. Another approach would be to use the strengths of every reconstruction and incorporate it into merged or combined models, which increase the correctness and the predictive value of a strain-specific model. Despite the vast amount of presented models in this review, there is no suitable model for every *S. aureus* strain available. Furthermore, missing annotations or identifiers that do not comply with the database identifier scheme impede the models’ re-usability and comparability. Standardization of all models would be desirable but is currently not feasible with the available tools without extensive manual labor for hundreds of identifiers. No omics data was incorporated into many of the published GEMs so far. Information about transcription profiles, for example, can help to refine metabolic reconstructions to better reflect the metabolic state of an organism in a defined environment. The incorporation of omics data can thus increase the predictive value of genome-based metabolic reconstructions^104^.

However, with the help of the already available reconstructions and further information, strain-specific models could be created or extended. Information from literature, merging of strain-specific models, and manual curation steps could further improve the predictive value of simulations and analyses of metabolic features of *S. aureus*. Having predictive GEMs can eventually lead to the identification of novel targets for antimicrobial therapies in the fight against antibiotic resistant strains of *S. aureus*.

## Supplementary information

Supplementary Information

## Data Availability

The availability of all models, including the improved models, is listed in the supplementary Table S1. The model collection was deposited in BioModels^105^ within COMBINE archive files (in OMEX format)^106^ and assigned the identifiers (1) MODEL2007110001, (2) MODEL2007150001, and (3) MODEL2007150002.

## Code availability

All the necessary scripts and resources for model modifications and improvements are available in a git repository at github.com/draeger-lab/S_aureus_GEMs_Collection.
