# Supplementary material for: Curating and comparing 114 strain-specific genome-scale metabolic models of Staphylococcus aureus
Source: NPJ Syst Biol Appl. 2021 Jun 29;7:30. doi: 10.1038/s41540-021-00188-4 (PMC8241996; doi:10.1038/s41540-021-00188-4)
Supplement: Supplementary file 1 — Supplementary Information [file 41540_2021_188_MOESM1_ESM.pdf]

Supplementary Material

Curating and Comparing 114 Strain-Specific Genome-Scale Metabolic Models of *Staphylococcus aureus*

Alina Renz[1,2,3] and **Andreas Dräger**[1,2,3,4,*]

[1]Computational Systems Biology of Infections and Antimicrobial-Resistant Pathogens, Institute for Bioinformatics and Medical Informatics (IBMI), University of Tübingen, 72076 Tübingen, Germany
[2]Department of Computer Science, University of Tübingen, 72076 Tübingen, Germany
[3]Cluster of Excellence 'Controlling Microbes to Fight Infections,' University of Tübingen, Germany
[4]German Center for Infection Research (DZIF), partner site Tübingen, Germany
[*]Correspondence: draeger@informatik.uni-tuebingen.de

Supplementary Table 1. Availability of all models in the *S. aureus* collection, including a direct link to their publication, and the model file(s). The link to the models refers to the most recent models. In the case of Lee *et al.*, the Path2Model project, and Bosi *et al.*, the models were extensively curated for this study and uploaded to the BioModels Database. The link between these three collections refers to the new version of the files. For all other models, the link points to the resource where the model was initially made available.

Model Count	Author	Year	Link to publication	Link(s) to model
1	Becker *et al.*	2005	identifiers.org/pubmed:15752426	identifiers.org/bigg.model:iSB619<br>identifiers.org/biomodels.db:MODEL1507180070
1	Heinemann *et al.*	2005	identifiers.org/pubmed:16155945	identifiers.org/biomodels.db:MODEL1507180072
13	Lee *et al.*	2009	identifiers.org/pubmed:17038190	identifiers.org/biomodels.db/MODEL2007150001
33	Path2Models project	2013	identifiers.org/pubmed:24180668	identifiers.org/biomodels.db/MODEL2007150002
64	Bosi *et al.*	2016	doi.org/10.1073/pnas.1523199113	identifiers.org/biomodels.db/MODEL2007110001
1	Magnusdóttir *et al.*	2017	doi.org/10.1038/nbt.3703	www.vmh.life/#microbe/Staphylococcus_aureus_subsp_aureus_USA300_FPR3757
1	Seif *et al.*	2019	identifiers.org/pubmed:30625152	identifiers.org/bigg.model:iYS854
